# Machine learning and deep learning for classifying the justification of brain CT referrals

**DOI:** 10.1007/s00330-024-10851-z

**Published:** 2024-06-24

**Authors:** Jaka Potočnik, Edel Thomas, Aonghus Lawlor, Dearbhla Kearney, Eric J. Heffernan, Ronan P. Killeen, Shane J. Foley

**Affiliations:** 1 University College Dublin School of Medicine, Dublin, Ireland; 2 University College Dublin School of Computer Science, Dublin, Ireland; 3 The Insight Centre for Data Analytics, Dublin, Ireland; 4 Mater Misericordiae University Hospital, Dublin, Ireland; 5 St. Vincent’s University Hospital, Dublin, Ireland; 6 Royal Victoria Eye and Ear Hospital, Dublin, Ireland

**Keywords:** Machine learning, Deep learning, Justification, Radiology, Clinical decision support

## Abstract

**Objectives:**

To train the machine and deep learning models to automate the justification analysis of radiology referrals in accordance with iGuide categorisation, and to determine if prediction models can generalise across multiple clinical sites and outperform human experts.

**Methods:**

Adult brain computed tomography (CT) referrals from scans performed in three CT centres in Ireland in 2020 and 2021 were retrospectively collected. Two radiographers analysed the justification of 3000 randomly selected referrals using iGuide, with two consultant radiologists analysing the referrals with disagreement. Insufficient or duplicate referrals were discarded. The inter-rater agreement among radiographers and consultants was computed. A random split (4:1) was performed to apply machine learning (ML) and deep learning (DL) techniques to unstructured clinical indications to automate retrospective justification auditing with multi-class classification. The accuracy and macro-averaged F1 score of the best-performing classifier of each type on the training set were computed on the test set.

**Results:**

42 referrals were ignored. 1909 (64.5%) referrals were justified, 811 (27.4%) were potentially justified, and 238 (8.1%) were unjustified. The agreement between radiographers (*κ* = 0.268) was lower than radiologists (*κ* = 0.460). The best-performing ML model was the bag-of-words-based gradient-boosting classifier achieving a 94.4% accuracy and a macro F1 of 0.94. DL models were inferior, with bi-directional long short-term memory achieving 92.3% accuracy, a macro F1 of 0.92, and outperforming multilayer perceptrons.

**Conclusion:**

Interpreting unstructured clinical indications is challenging necessitating clinical decision support. ML and DL can generalise across multiple clinical sites, outperform human experts, and be used as an artificial intelligence-based iGuide interpreter when retrospectively vetting radiology referrals.

**Clinical relevance statement:**

Healthcare vendors and clinical sites should consider developing and utilising artificial intelligence-enabled systems for justifying medical exposures. This would enable better implementation of imaging referral guidelines in clinical practices and reduce population dose burden, CT waiting lists, and wasteful use of resources.

**Key Points:**

- *Significant variations exist among human experts in interpreting unstructured clinical indications/patient presentations.*
- *Machine and deep learning can automate the justification analysis of radiology referrals according to iGuide categorisation.*
- *Machine and deep learning can improve retrospective and prospective justification auditing for better implementation of imaging referral guidelines.*

## Introduction

Diagnostic imaging plays a pivotal role in current medical care, allowing fast and accurate diagnosis, treatment, and surveillance of a range of conditions. However, overuse of imaging is commonplace, resulting in downstream cascades of more imaging, higher healthcare expenses, increased patient radiation exposure, and potential harm [1, 2]. The worldwide volume of diagnostic imaging, particularly computed tomography (CT) and magnetic resonance imaging (MRI), has significantly increased, leading to a rise in the rate of unjustified, low-value examinations [3–7]. Moreover, the Food and Drug Administration estimates that 20–50% of CT scans in the US are unnecessary [8]. This raises concerns, as CT contributes more than 60% of the collective effective dose from imaging studies, although accounting for less than 10% of all procedures [9].

Currently, available approaches addressing the excessive utilisation of CT include the introduction of diagnostic imaging pathways, providing alternative imaging, specialist involvement, post-imaging audit, and feedback where each referrer’s ordering frequency is compared to the departmental average [10]. Numerous barriers lessen the impact that clinical imaging guidelines have on the frequency of low-value scans. These barriers encompass financial reasons, equipment availability, lack of familiarity with guidelines, workplace culture, the fear of “missing something”, self-referrals, patient expectations, and, most notably, inefficient implementation [11–13].

Regular retrospective justification auditing [14] can address guideline compliance and monitor the impact of quality improvement. Nevertheless, many institutions find it challenging to conduct regular retrospective auditing due to constraints on time, staff, and budget. A more reliable real-time justification process and clinical decision support (CDS) for implementing guidelines in clinical routine [15] is desirable.

Two studies demonstrated the feasibility of automated justification analysis of radiology referrals by applying natural language processing (NLP) to unstructured clinical indications, machine learning (ML), and deep-learning (DL) techniques for binary classification: justified or unjustified. In the first study [16], researchers conducted a manual retrospective justification audit of 375 brain CT referrals using iGuide and merged unjustified and potentially justified referrals due to dataset limitations. The second study [17] involved the justification analysis of 1020 lumbar spine MRI referrals from two clinical sites, where the data was labelled based on clinical experience.

Considering the upward trend in the utilisation of diagnostic imaging and the persisting issue of inappropriate CT imaging, as well as the limitations in previous studies, an important aspect of our study was the development of an artificial intelligence (AI)-based iGuide (Quality and Safety in Imaging GmbH, Vienna, Austria) interpreter on real-world data to enable efficient implementation of justification standards in radiology practice. We aimed to determine its generalisability across multiple clinical sites for the most frequent CT scan nationally, brain CT [18], and compare human experts with prediction models.

## Methods

The study was granted an ethics exemption by our institution’s research ethics committee (REERN: LS-E-21-216-Potocnik-Foley). Each participating clinical site carried out a data protection impact assessment and exempted the study from a full ethics review. The anonymised, encrypted data was stored in Excel format on a university cloud storage. Python (version 3.8.16) was used to perform data science tasks.

### Data sourcing and labelling

Three tertiary referral hospitals, two private and one public, provided anonymised referrals of all brain CT scans performed on adult patients in 2020 and 2021. The electronically extracted referrals included information on patient gender, age, and unstructured clinical indications.

Referrals from each clinical site were randomly shuffled and divided into multiple groups, each consisting of approximately 1000 referrals. One group from each site was then randomly selected. Duplicate and inadequate referrals containing no clinical indications were excluded. Two radiographers (6 and 8 years of experience) analysed the justification of the selected referrals. Subsequently, two consultant radiologists (11 and 15 years of experience) analysed the referrals where the two radiographers disagreed. Each clinical site was equally represented in the dataset. The annotators used iGuide clinical imaging guidelines to assist their decision-making when manually justifying referrals. Specifically, a cloud-based platform for structured referring, xWave CDS [19], enabled seamless access to iGuide recommendations for a specific clinical indication identified by humans. The vetting pipeline, utilising xWave CDS (xRefer), is described in our previous study [16]. The final justification label for each referral was determined by a majority vote, with consensus being reached in case of a tie or lack of clinical evidence.

For referrals deemed unjustified or potentially justified, alternative imaging methods were proposed based on iGuide. Inter-rater agreement among the radiographers and consultants was computed to determine if significant variations in interpreting unstructured clinical indications exist.

### Data preprocessing and learning

Unstructured clinical indications were tokenized using the word tokenizer from the Natural Language Toolkit platform. The tokens were then normalised, and special characters, except for “#”, were ignored. The next step involved spell-checking with the Enchant algorithm with a custom medical dictionary comprising medical terms found in our dataset. Any errors in the spell-checking outputs were mapped to the correct terms. English and non-clinical stop words, except for negations, were also filtered. Common abbreviations were spelled out. Our data cleansing was aimed at developing a natural language processor capable of handling real-world clinical text of varying quality. This approach was chosen instead of creating “ideal lab data”, which could have negatively impacted the original data quality and posed legal challenges in the context of medical device law [20]. Finally, we vectorised the free-text with the bag-of-words (BoW) and term frequency-inverse document frequency (TF-IDF) vectorizers from the Scikit-learn library. Additionally, a pretrained clinical Word2vec model [21] was used to generate sentence-level vectors by averaging the word vectors of words present in each referral.

Prior to text preprocessing and feature extraction, the multi-class dataset was randomly undersampled to the minority class. The undersampled dataset was randomly split into stratified training and test sets (4:1). Each prediction model’s hyperparameters were tuned on the training set through GridSearch with 10-fold cross-validation while considering minimum document frequencies between two and five to ignore infrequent terms and improve model effectiveness and efficiency. ML algorithms included were support vector machine, logistic regression, and gradient-boosting ensemble. Models of each type with the highest training accuracy were evaluated on the test set. Keras and Tensorflow libraries were used to train multilayer perceptron (MLP) and bi-directional long short-term memory (Bi-LSTM) networks. The outputs of the best-performing classifier were compared to the consistency of the radiographers’ justification analysis using the test set.

**Fig. 1:**
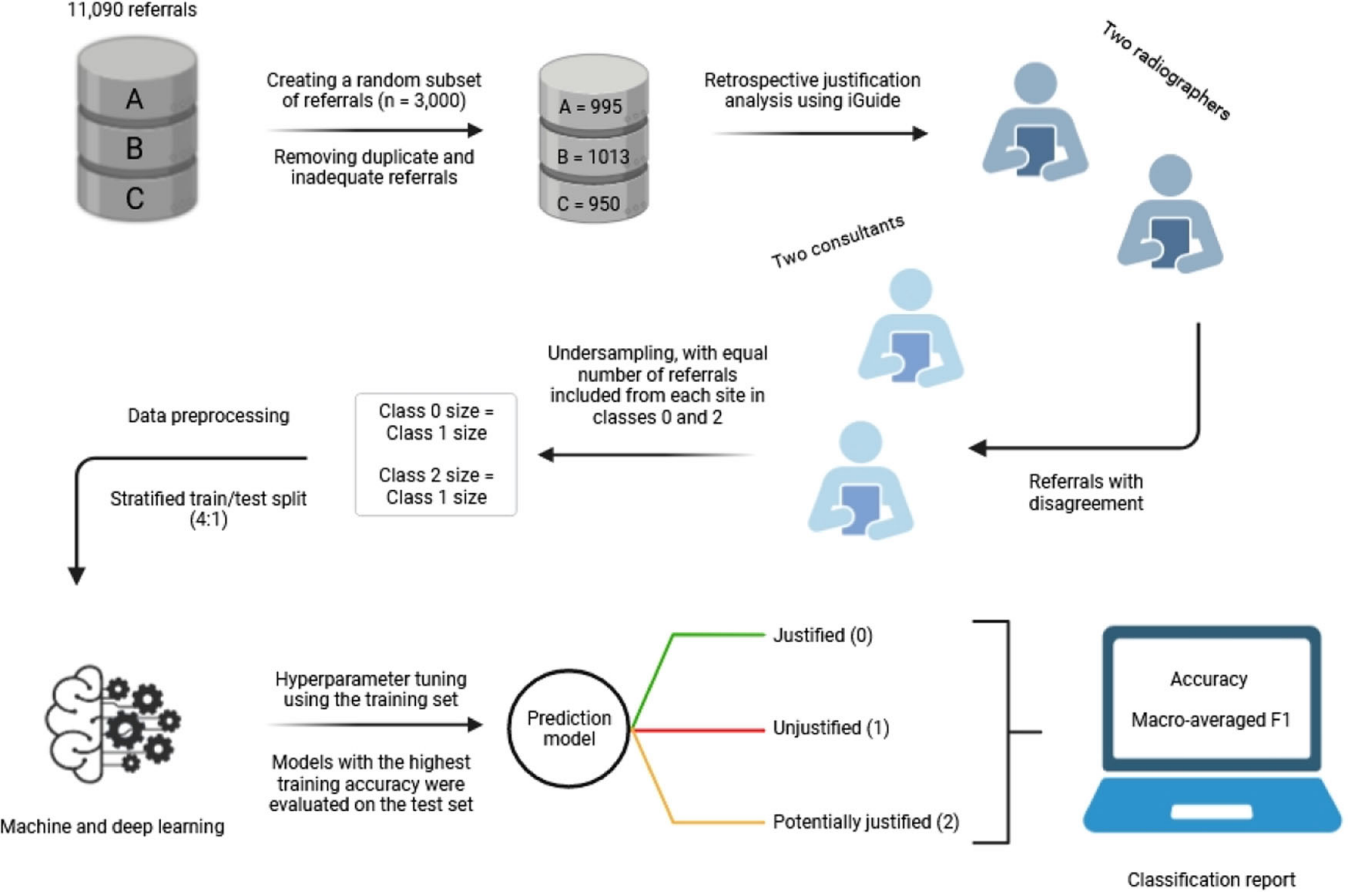
Data analysis workflow (created with Biorender)

**Fig. 2:**
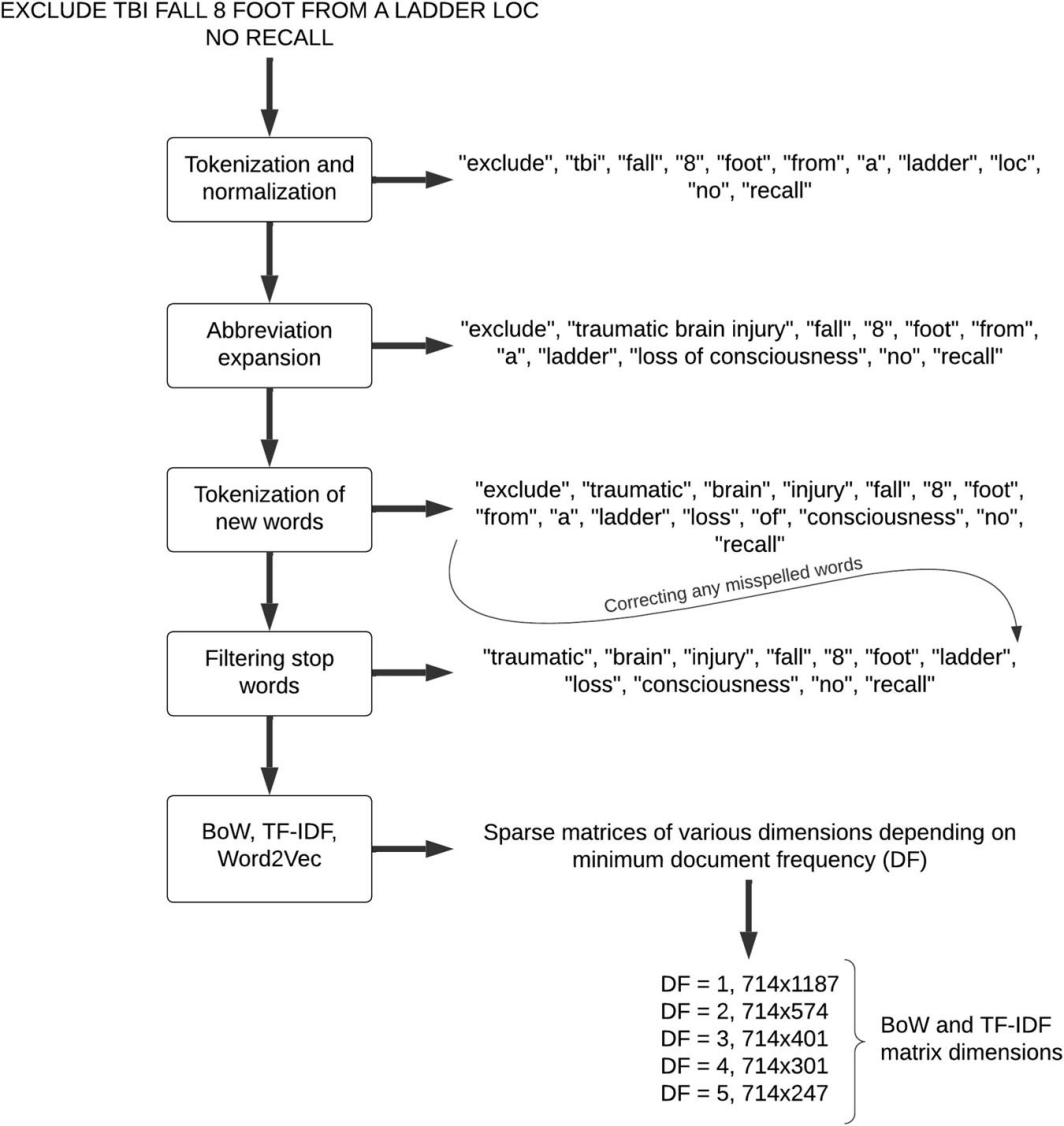
Sample referral flow through our NLP pipeline. Varying sizes of input dimensions (training + test sets) for prediction models are outlined

The Bi-LSTM was chosen as the sole DL model in our study due to its superior performance in classifying free-text radiology referrals and data efficiency compared to convolutional neural networks [17]. Its embedding layer transformed each word into a 128-dimensional vector. Input sequences were padded to have an input length of 50. The model featured one Bi-LSTM layer with 100 LSTM units. The training process halted when the loss on the training set ceased to decrease − patience = 2. Training epochs were set to 50. MLP models had one hidden layer with 16 neurons. Both the Bi-LSTM and MLP output layers comprised three neurons, employing the softmax activation function. The single hidden layer of the MLPs used either a sigmoid or rectified linear unit. Each MLP model was manually optimised over 30 epochs. Smaller batch sizes of 16 were employed for Bi-LSTM and MLP training. The Bi-LSTM and MLP models were compiled with the Adam optimiser and categorical cross-entropy. Accuracy and the unweighted, harmonic mean of precision and recall (F1) for each label were computed for each classifier. The approach to analysing the data is summarised in Fig. 1. A preprocessing flow of a single instance through our NLP pipeline, along with the associated outputs, is illustrated in Fig. 2.

## Results

### Retrospective justification audit

11,090 referrals were initially collected: 2651 from site A, 4351 from site B, and 4088 from site C. Since 42 referrals were disregarded, the justification analysis included 2958 referrals. Two radiographers disagreed on 946 (32.0%, *κ* = 0.268) referrals, which were subsequently analysed by the two consultants. 839 (88.7%) referrals were labelled on the basis of a majority vote. For the remaining referrals (*n* = 107, 11.3%), consensus was reached. Of the 946 referrals analysed, the radiologists disagreed on 274 (29.0%, *κ* = 0.460).

**Fig. 3:**
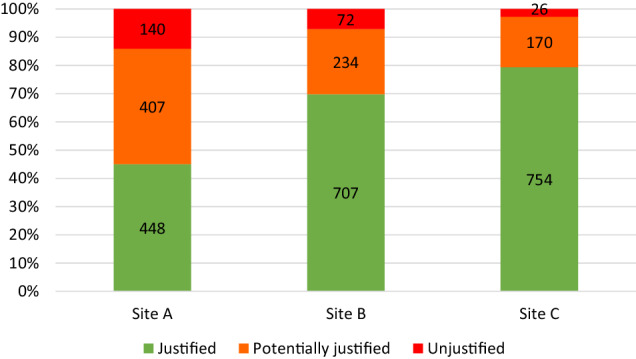
Justification outcomes associated with each clinical site

A detailed breakdown of the justification rates is demonstrated in Fig. 3. Site A, a private facility, had the lowest rate of justified scans (*n* = 448, 45.0%) followed by site B (*n* = 707, 69.8%) and the public site C (*n* = 754, 79.4%). Overall, 238 (8.1%) referrals were unjustified with sites A and B contributing 140 (58.8%) and 72 (30.3%) referrals, respectively. 811 (27.4%) referrals were deemed potentially justified, with the majority (*n* = 641, 79.0%) coming from sites A and B.

Common clinical indications found in our dataset that are inappropriate for imaging with CT include dizziness/vertigo, long-lasting headaches without new features, tinnitus, and recurrent falls. Potentially appropriate referrals included migraine-like symptoms, oncology-related indications, space-occupying lesions, blackout episodes or syncope/fainting, and slowly progressing ataxia. Patient presentations consistent with a stroke/transient ischemic attack, head injury, headache, or subdural haematoma after trauma, sudden thunderclap/”worst of life” headache, and intracranial/intracerebral haemorrhage justify a CT brain [22]. The most common alternative imaging modality identified was MRI, which could have replaced 157 (66.0%) unjustified and 450 (55.5%) potentially justified CT examinations. An abdominal ultrasound would have been more appropriate for eight (3.4%) unjustified referrals querying the source of nausea and/or vomiting without providing any information on ultrasound-related findings. In total, only three referrals contained information on MRI availability or contraindications. For the remainder of unjustified and potentially justified referrals, MRI scored either lower or equal to CT, or there were no iGuide recommendations available.

### Machine and deep learning

**Fig. 4:**
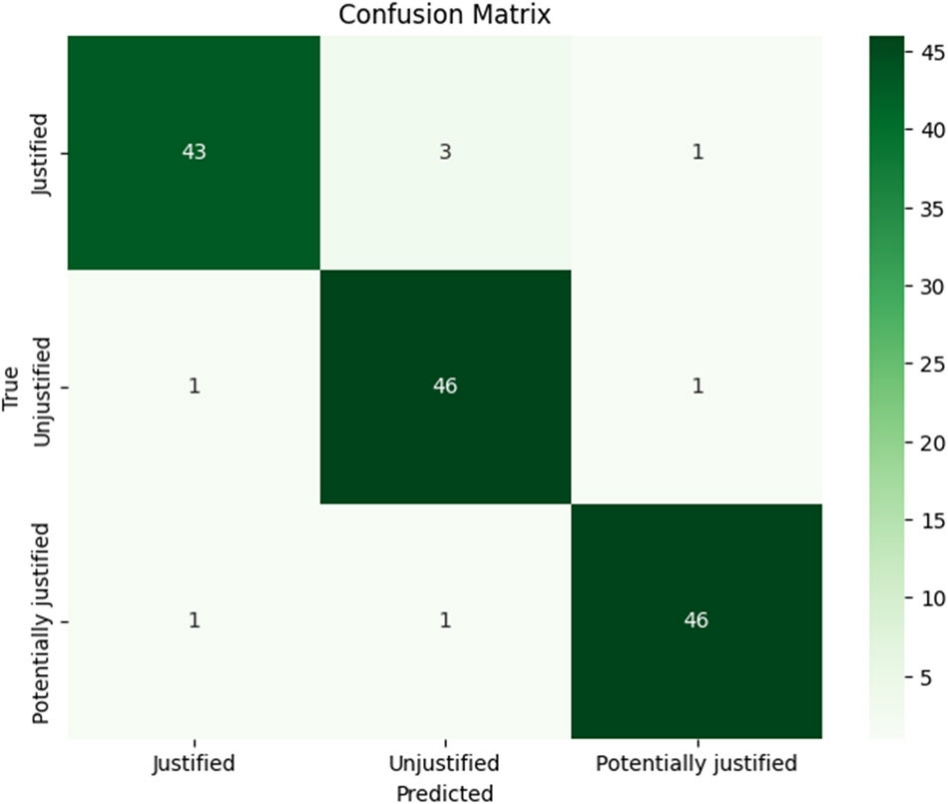
Confusion matrix of the best-performing gradient-boosting classifier

The minority class comprised 238 unjustified referrals, hence the remaining two classes were randomly undersampled to match the size of the minority class. Different combinations of feature extraction techniques and multi-class classifiers were evaluated on the test set. Performance metrics for each classifier are demonstrated in Table 1. Gradient-boosting classifier, in combination with BoW embeddings, achieved a superior performance resulting in 94.4% accuracy and a macro F1 of 0.94, with predictions visualised in Fig. 4. Considering the test set, radiographers disagreed on 69 (48.3%) referrals, whereas the superior classifier made only eight (5.6%) false predictions. Sample ambiguous and misclassified referrals are included in Table 2.

**Table 1:** Multi-class classifier evaluation metrics on training and test sets

Bag-of-words				
	Training set		Test set	
Prediction model	Accuracy (%)	Macro F1	Accuracy (%)	Macro F1
SVM	91.2	0.91	91.6	0.92
LR	95.6	0.96	92.3	0.92
GBC	99.3	0.99	94.4	0.94
MLP	99.3	0.99	90.9	0.91

Term frequency-inverse document frequency				
SVM	100.0	1.00	93.7	0.94
LR	94.2	0.94	89.5	0.90
GBC	100.0	1.00	93.0	0.93
MLP	94.4	0.94	90.9	0.91

Word2Vec (BioWordVec)				
SVM	96.2	0.96	90.2	0.90
LR	95.6	0.96	89.5	0.90
GBC	100.0	1.00	88.1	0.88
MLP	90.9	0.91	90.2	0.90
Bi-LSTM	98.8	0.99	92.3	0.92

*SVM* support vector machine, *LR* logistic regression, *GBC* gradient-boosting classifier, *MLP* multilayer perceptron, *Bi-LSTM* bi-directional long short-term memory

**Table 2:** Sample ambiguous and misclassified referrals by the best-performing classifier

Ambiguous referrals	
“Headaches with episodes of collapse. ?cause”	
“80 years old lady presenting with intermittent vertigo + persyncopal episodes. One episode of altered sensation, facial, left-sided. bg of htn and hyperlipidemia. ? Intracerebral cause”	

Justified referrals misclassified as unjustified	
“Seizures, o/e diplopia b/l temporal quadrants”	
“Admitted following a fall and influenza extremely agitated and confused need extra sedation for ct brain to r/o bleeding/assess for cerebral oedema”	
“81-year-old female admitted with unwitnessed fall. bg dementia, found on ground. Large bruise over right forehead. On aspirin.”	

Unjustified referrals misclassified as potentially justified	
“Headaches”	

Unjustified referrals misclassified as justified	
“19-year-old boy large bump bruise over mid forehead Impression probable superficial haematoma and forehead”	

The second-best-performing model was the TF-IDF-based support vector machine, which achieved 93.7% accuracy and an identical macro F1 score. DL approaches were less effective with Bi-LSTM achieving 92.3% accuracy and a macro F1 of 0.92, resulting in higher performance compared to the MLP models achieving a 90% accuracy and a macro F1 between 0.90 and 0.91, regardless of the text preprocessing method.

## Discussion

Justification is the cornerstone of radiation protection and is legally mandated in many countries [4], but is likewise essential for the appropriate use of valuable healthcare resources while optimising patient diagnostic and treatment pathways. Current evidence, including the outcomes of our justification audit, points to substantial rates of inappropriate imaging, which require further efforts, especially in CT due to its large contribution to population dose. Sites A and B, the two private institutions included in our justification analysis, had lower rates of justified scans compared to the public site C. This raises questions about the vetting processes, particularly in site A, and supports claims that private facilities may prioritise a higher volume of outpatient scans for financial reasons, resulting in more unjustified imaging [23]. In general, the results of the study show that our methods based on ML and DL can automate the iGuide justification analysis of CT referrals and generalise on multi-institutional data. The prediction models used the unstructured clinical indications as the input with justification outcomes serving as target features for multi-class classification. More efficient approaches, involving ML and DL-based NLP, can help streamline the current referral vetting process, which is manual, highly unstructured, costly, and most importantly, inefficient. In addition, since prediction models can analyse thousands of referrals within seconds, large-scale retrospective auditing would become feasible for the majority of stakeholders. It is worth noting that the referrals included in our study pertain to CT scans that have already been performed and were vetted by referring clinicians and radiographers. The fact that ML and DL outperformed both the clinical staff and human experts in vetting referrals suggests that a “second pair of eyes” could prove beneficial when manually interpreting unstructured patient presentations. Leveraging NLP and prediction models could, therefore, assist in justifying medical exposures, as well as interpreting unstructured clinical indications. As a result, the implementation of retrospective and potentially prospective CDS would enable better integration of referral guidelines (e.g., iGuide) into clinical practices, ensuring consistency across various clinical sites.

The structure, style, and quality of radiology referrals vary significantly amongst referrers and institutions [16, 24]. They may contain slang words, uncommon abbreviations, misspellings, and either very brief or overly detailed clinical history. Expressions that convey doubt or uncertainty, and query multiple pathologies are also present, making them ambiguous and complex to interpret. This variability was demonstrated during the retrospective justification audit where both radiographers and consultants disagreed on a statistically significant portion of referrals analysed. The ambiguous referrals in Table 2 illustrate the complexity and possible interpretations. A very first consideration would be determining whether “collapse” corresponds to syncope/fainting with or without a transient or total loss of consciousness. Furthermore, a combination of headaches with collapse makes the interpretation more challenging. iGuide contains three structured indications that are consistent with the patient presentation:

1. A headache due to collapse and subsequent head trauma—justified.
2. Migraine (basilar type)—no recommendations, potentially justified.
3. Altered level of consciousness without known cause—justified.

Likewise, in the second referral, it is unclear whether the CT scan was requested due to vertigo, presyncopal episodes, or a subjective altered facial sensation. The clinical history indicates that the patient had hypertension and hyperlipidaemia, suggesting a potential underlying stroke. Interestingly, in both cases, the radiographers disagreed, and so did the consultants.

The problem of imbalanced datasets is noticeable in classification tasks, as it degrades classification performance [25]. There are considerations with regard to the BoW- and TF-IDF-based models outperforming the Word2Vec and DL approaches. Given that our dataset was limited in size due to undersampling, our Word2Vec and DL models were more prone to overfitting and yet achieved > 90% accuracy on the test set. MLPs with more than three hidden layers were significantly overfitting on the training data. A similar observation was noted with the Bi-LSTM classifier when building a deep architecture, indicating a natural limitation in our dataset. Initially, when developing the model on the imbalanced dataset consisting of 2958 referrals, the bias towards the majority class needed to be addressed. The initial undersampling to the second largest class failed to yield significantly better results. Further undersampling to the minority class helped overcome the bias. Although one reasonable approach to addressing the class imbalance would be synthetic oversampling, there is no unanimity on the best, most efficient, and representative technique in clinical text classification. For example, the word-swapping technique was used to produce synthetic radiology referrals [17], which lack context and are not sufficiently realistic. Authors in this study report nearly 100% training and test accuracy when classifying MRI lumbar spine referrals as justified or unjustified, which is difficult to achieve on real data. Similarly, synthetic minority oversampling (SMOTE) can be used to generate synthetic examples via interpolation between text features of the original example and its random *k*-nearest neighbours [26]. SMOTE tends to be less efficient when the feature distributions of the classes overlap, which is the case in unstructured referring practices as discussed below. There are several other synthetic oversampling techniques demonstrating promising results, including generative adversarial networks [27]. However, their usability in the referring context and output similarity with the real data needs to be investigated.

The BoW-based gradient-boosting classifier classified three justified referrals as unjustified. Only two referrals, which were deemed justified, contained the word “diplopia” in our undersampled dataset. These two referrals were included in the test set. Additionally, only six referrals contained the word “seizures”, which is associated with all three classes making it less discriminative. Four of these were included in the training set. This suggests that the model randomly assigned a label to the first referral. The same pattern was observed for the remaining two referrals, which had a combination of ambiguous features, highlighting the need for contextual predictions. For instance, a bruise from a fall typically does not require imaging. However, if the patient is on anticoagulation therapy, it is reasonable to consider the possibility of an intracranial bleed.

The prediction model classified one unjustified referral as potentially justified. In our dataset, the term “headaches” is often surrounded by terms suggesting long-term, migraine-like features, which potentially justify CT imaging. In this particular case, there was no additional information provided, leading the human raters to assume that the patient was experiencing chronic headaches without new features, which does not warrant imaging.

There was one unjustified referral classified as justified. The word ‘haematoma’ is strongly associated with “cerebral” and “subdural” in our dataset and justifies CT imaging in nearly all cases. However, because word embeddings lack context, the model failed to distinguish the nature of the haematoma. The term “superficial” appeared only once in our undersampled dataset.

In general, our study would have benefited from a larger, balanced, real dataset to demonstrate the advantages of DL and transfer learning. Ideally, model tuning should be performed on a validation set to guard from overfitting and ensure robustness. Nonetheless, all classifiers exhibited impressive overall accuracy and strong iGuide generalisation capabilities. As imaging referrals typically include only a brief synopsis of the patient’s clinical information, it can be difficult to understand the physician’s decision-making without a full review of the patient’s health record. Since our dataset lacked information on the source of referrals, it was unfeasible to perform context comparisons. The clinical sites involved here request generic brain CT for related referrals, with specific protocolling (i.e., non-contrast, contrast-enhanced, angiography, etc.) performed by the radiology team on review of the provided clinical information, hence training separate prediction models for specific types of brain CT was unfeasible.

Further work is needed to delve into transfer learning in more detail and train a large language model specific to the referring language to showcase the superiority of contextual embeddings. A large-scale prediction model training would address the gap that currently exists. Future research should consider investigating the value of NLP in interpreting unstructured clinical indications. This would enhance collaboration between human radiologists, who contribute content knowledge and the ability to find near-optimal solutions, and AI, which provides knowledge based on available training samples. Ideally, these should be numerous and diverse to cover all variants, aiming for better augmentation rather than as a replacement for human experts [28]. It is vital to address practical principles prior to clinical implementation to overcome the ethical and legal issues associated with such applications [29]. Including other types of CT scans and modalities would be the next reasonable step to consider.

In conclusion, the overuse of diagnostic imaging, in particular CT, is resulting in a substantial number of unjustified examinations. ML and DL have the potential to streamline the justification process, generalise across multiple clinical sites, and assist referrers in choosing appropriate diagnostic imaging. Therefore, this makes large-scale retrospective and prospective vetting of radiology referrals feasible. Consequently, the efficiency and usage of the existing support tools, such as clinical referral guidelines, can be improved via AI approaches.

## Abbreviations

AI: Artificial intelligence
Bi-LSTM: Bi-directional long short-term memory
BoW: Bag-of-words
CDS: Clinical decision support
CT: Computed tomography
DL: Deep learning
ML: Machine learning
MLP: Multilayer perceptron
MRI: Magnetic resonance imaging
NLP: Natural language processing
TF-IDF: Term frequency-inverse document frequency
SMOTE: Synthetic minority oversampling technique
